# Atherogenic *ω*-6 Lipids Modulate PPAR- EGR-1 Crosstalk in Vascular Cells

**DOI:** 10.1155/2011/753917

**Published:** 2011-10-26

**Authors:** Jia Fei, Carla Cook, Miriah Gillespie, Bangning Yu, Khyra Fullen, Nalini Santanam

**Affiliations:** ^1^Department of Pharmacology, Physiology and Toxicology, Joan C. Edwards School of Medicine, Marshall University, Huntington, WV 25755, USA; ^2^Department of Pharmacology and Experimental Therapeutics, LSU Health Sciences Center, New Orleans, LA, USA

## Abstract

Atherogenic **ω**-6 lipids are physiological ligands of peroxisome proliferator-activated receptors (PPARs) and elicit pro- and antiatherogenic responses in vascular cells. The objective of this study was to investigate if **ω**-6 lipids modulated the early growth response-1 (Egr-1)/PPAR crosstalk thereby altering vascular function. Rat aortic smooth muscle cells (RASMCs) were exposed to **ω**-6 lipids, linoleic acid (LA), or its oxidized form, 13-HPODE (OxLA) in the presence or absence of a PPAR**α** antagonist (MK886) or PPAR**γ** antagonist (GW9662) or PPAR-specific siRNA. Our results demonstrate that **ω**-6 lipids, induced Egr-1 and monocyte chemotactic protein-1 (MCP-1) mRNA and protein levels at the acute phase (1–4 hrs) when PPAR**α** was downregulated and at subacute phase (4–12 hrs) by modulating PPAR**γ**, thus resulting in altered monocyte adhesion to RASMCs. We provide novel insights into the mechanism of action of **ω**-6 lipids on Egr-1/PPAR interactions in vascular cells and their potential in altering vascular function.

## 1. Introduction

Early growth-response (Egr-1) is a critical mediator of vascular pathology by activating its dependent genes, tumor necrosis factor-*α* (TNF*α*) (a potential stimulator of nuclear factor kappa B-NF*κ*B) and monocyte chemotactic protein-1 (MCP-1) [1–4]. MCP-1 has a specific Egr-1 binding element in its promoter region and is therefore directly activated by Egr-1 [5, 6]. Many of these target genes in turn activate Egr-1 by a positive feedback mechanism and thereby further amplifying their effects [7]. 

Peroxisome proliferator-activated receptors (PPAR*α*, *β*, *γ*) are transcription factors that regulate various cellular processes including lipid and glucose homeostasis [8, 9]. Both PPAR*α* and PPAR*γ* are expressed in vascular cells including endothelial cells, smooth muscle cells (vSMCs), and monocyte/macrophages [10, 11]. Activators of PPARs have beneficial effects against atherosclerosis [12, 13]. Ligand-mediated activation of PPAR*α* [14–17] and PPAR*γ* attenuates the release of inflammatory factors including the production of monocyte chemotactic protein-1 [18, 19]. Interestingly, PPARs can directly interact with Egr-1 and attenuate its downstream effects [20]. This attenuation is effective by the activation of both PPAR isotypes, PPAR*α* [21] and PPAR*γ* [22]. However, in vascular cells there exists a time-dependent crosstalk between Egr-1 and PPARs, for example, Egr-1 exhibits a critical early stimulatory effect but a later inhibitory effect on PPARs [23]. 

Atherogenic *ω*-6 lipids such as linoleic acid (LA, 18 : 2*n* − 6) and its oxidized forms, 13-hydroperoxy octadecadienoic acid (13-HPODE) and 13-hydroxyoctadecadienoic acid (13-HODE) are physiological ligands for both PPAR*α* [24] and PPAR*γ* [25–28], that can covalently interact with PPARs and alter their activity [29, 30]. The biological significance of these interactions is not well understood. LA is the predominant polyunsaturated fatty acid found in the Western diet [31], which at lower doses has lipid-lowering beneficial effects [32], but, deleterious effects when consumed in excess [33]. Oxidized forms of LA (oxidized linoleic acid (OxLA)) and other oxidized *ω*-6 lipids are present in significant amounts in heated oils and processed foods [34–37]. *ω*-6 lipids are also major components of oxidized low-density lipoproteins (Ox-LDLs) that exhibit both pro- and anti-atherogenic effects on vascular cells [38–40].

**Scheme 1:**
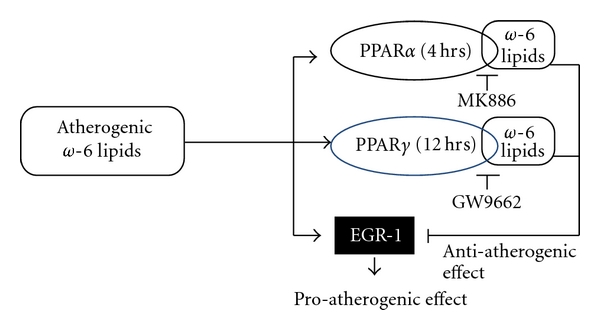
Atherogenic *ω*-6 lipids modulate PPAR-Egr-1 crosstalk. A schematic representation of a possible mechanism by which *ω*-6 lipids and their oxidized forms regulate PPAR-Egr-1 crosstalk in a time-mediated fashion and thereby altering smooth muscle cell function. *ω*-6 lipids seemed to have a time-dependent modulation of PPAR isotypes, PPAR*α* at acute phase and PPAR*γ* at subacute phase. This modulation of PPAR isotype altered the ability of these lipids to exert an antiatherogenic effects via PPARs or proatherogenic effects via Egr-1.

Since Egr-1 regulates the proinflammatory and PPARs regulates the anti-inflammatory pathways in vascular cells, and that atherogenic *ω*-6 lipids interact with both these transcription factors, we speculate that these lipids could influence the crosstalk between these two transcription factors in a time- and concentration-dependent manner (Scheme 1) and thereby influence atherogenic events. In this study, the ability of LA and OxLA (*ω*-6 lipids that are abundant in dietary sources and OxLDL) to influence Egr-1 and PPAR crosstalk was investigated in primary rat aortic smooth muscle cells (RASMCs) in the presence or absence of known PPAR antagonists or by knocking down PPARs by siRNA approach. To our knowledge this is the first study that has investigated the ability of atherogenic *ω*-6 lipids to modulate EGR-1-PPAR crosstalk. Our findings will provide insights into the biological significance of the interactions between physiological ligands of PPAR and other transcription factors.

## 2. Materials and Methods

### 2.1. Materials

Dimethylsulfoxide (DMSO), PPAR*γ* antagonist (GW9662) was obtained from Invitrogen (Carlsbad, CA). PPAR*α* antagonist (MK886) was obtained from Cayman (Ann Arbor, MI). Linoleic acid and soybean lipoxidase were obtained from Sigma (St. Louis, MO). Rabbit Egr-1 and MCP-1 monoclonal antibody was obtained from Abcam (Cambridge, MA). Rabbit anti-actin monoclonal antibody was obtained from Sigma (St. Louis, MO). PPRE-luciferase construct [p(AOX_3_)-TKSL] was a gift (Dr. Richard Niles, Marshall University, Huntington, WV).

### 2.2. Oxidation of Linoleic Acid

A 10 mM stock solution of linoleic acid (LA-18 : 2) was first prepared in absolute ethanol which was further diluted in phosphate-buffered saline (PBS) to make 0.1 mM LA solution. A fresh aliquot of 0.1 mM LA solution was oxidized with soybean lipoxidase (100–200 U/100 nmol, 1 hr at 37°C) to generate oxidized linoleic acid (OxLA-13-HPODE and 13-HODE) [41]. The conversion of LA to OxLA (HPODE or HODE) was monitored spectrophotometrically (Shimadzu, Columbia, MD) as an increase in the absorbance at optical density of 234 nm. Usually, >98% of unoxidized LA was converted to OxLA.

### 2.3. Cell Treatment and Sample Collection

Primary rat aortic smooth muscle cells (RASMCs) were cultured in specific growth media following the recommendations of the manufacturer (ATCC, Manassas, VA) and used at a passage number below 15. Unless otherwise indicated, 70–80% quiescent cells were first pretreated with GW9662 (1 *μ*M), MK886 (10 *μ*M) or DMSO (1 *μ*M) (vehicle) for 2 hours. Pretreated cells were then exposed to either LA or OxLA at 10, 25 and 50 *μ*M concentrations, for 0, 1, 4, or 12 hours (hrs). The control (CTRL) was defined as cells treated with vehicle alone (DMSO). Each treatment was run in duplicates and one set of cells were used for qRT-PCR analyses and the second set was used for Western blotting. Each experiment was repeated at least three times.

### 2.4. siRNA Transfection

RASMCs were cultured to 50–70% confluence and then transfected using 50 nmoles of a pooled mixture of ON-TARGETplus SMARTpool siRNA duplexes (SMARTpool, Thermo Scientific Dharmacon, Lafayette, CO) for PPAR*α*, PPAR*γ* or a nonspecific control siRNA (Nontargeting pool, Thermo Scientific Dharmacon, Lafayette, CO) using Thermo Scientific DharmaFECT transfection reagents and siRNA transfection protocol (Thermo Scientific Dharmacon, Lafayette, CO). Forty eight hours after transfection, quiesced cells were treated with vehicle (CTRL), 25 and 50 *μ*M LA, and 10, 25 and 50 *μ*M OxLA for either 4 or 12 hours. Egr-1 mRNA levels were determined after each treatment by real-time PCR.

### 2.5. Quantitative Real-Time Reverse-Transcriptase PCR (qRT-PCR)

Total RNA was extracted from the treated cells using the TRIzol reagent kit (Sigma, St-Louis, MO) according to the manufacturer's protocol. The mRNA levels of Egr-1 and MCP-1 were analyzed in a MyiQ real time PCR system (Bio-Rad, Hercules, CA). *β*-actin was used as the house-keeping gene. The real-time PCR was carried out in 25 *μ*L of a SYBR green reaction mixture containing 1 *μ*L of cDNA and iQ SYBR Green Supermix (Bio-Rad, Hercules, CA) containing the respective primers: Egr-1 (NM_012551) 5′-aacactttgtggcctgaacc-3′, 3′-aggcagaggaagacgatgaa-5′; MCP-1 (NM_031530) 5′-atgcagttaatgccccactc-3′, 3′-ttccttattggggtcagcac-5′; *β*-actin was used as the house-keeping control (NM_031144) 5′-gtccacccgcgagtacaacct-3′, 3′-tcgacgacgagcgcagcgata-5′. A sequence detection program calculated a threshold cycle number (CT) at which the probe cleavage-generated fluorescence exceeded the background signal [42]. The real-time PCR results were expressed as fold change ± Standard Error of ΔCt for each group compared to control (vehicle treatment) after normalizing to beta actin (housekeeping gene) using the Pfaffl method (2^−ΔΔCt^) [42].

### 2.6. Western Blot Analysis

For Western blotting, the treated cells were rinsed in phenol-red free Hanks buffer and whole cell lysates were prepared in RIPA buffer (TRIS 50 mM, sodium chloride 150 mM, Triton 1%, sodium deoxycholate 1%, SDS 0.1%, EDTA 5 mM) containing protease inhibitors (Roche Diagnostics, Indianapolis, IN). Total protein in the cell lysates was quantified using the Lowry method [43]. Equal amount of the cell proteins were subjected to SDS-PAGE. After transfer, blots were probed individually with a solution of rabbit antibody to rat Egr-1 (1 : 3000), MCP-1 (1 : 7000), PPAR*α* (1 : 2000), PPAR*γ* (1 : 7000), or *β*-actin (1 : 1000) as housekeeping protein and then analyzed using the chemiluminescence detection method (Millipore, Billerica, MA). The protein levels were quantified by densitometry of the respective bands on the autoradiograph (Bio-Rad, Hercules, CA). The results were expressed as the ratio of protein levels in treated samples compared to CTRL after normalizing to *β*-actin.

### 2.7. Transient Transfection and Luciferase Reporter Assay

RASMCs in 12-well plates (50,000 cells per well) were transfected with 0.5 *μ*g per well of the PPRE-luciferase construct (p(A-OX3)-TKSL) using Lipofectamine-2000 transfection reagent (Promega, Madison, WI). After 24 hrs of transfection, cells were transferred to serum-free EMEM media containing 1% charcoal stripped fetal bovine serum and either pretreated or untreated with PPAR antagonists, [MK886 (10 *μ*M) or GW9662 (1 *μ*M)]. Following pretreatment, the cells were exposed for 4 hrs with either LA or OxLA at 10, 25, 50 *μ*M concentrations. Controls (CTRL) were defined as samples without LA or OxLA treatment. At the end of lipid treatment, the cells were washed in PBS three times and solubilized in 1X lysis buffer (Roche, Indianapolis, IN). PPRE transactivity was determined in the cell lysates by assaying for firefly luciferase activities using the Luciferase Reporter Assay System (Berthold, Germany). Each experiment was performed in duplicates and repeated three times. The results were expressed as the ratio of relative luciferase units (RLU) in treated samples/CTRL values.

### 2.8. Monocyte Adhesion Assay

To demonstrate the physiological consequences of alterations in Egr-1/MCP-1 levels by *ω*-6 lipids on vascular function, monocyte adhesion studies were performed using established protocols [44]. Briefly, RASMCs were seeded in 12-well plates at a cell density of 1 × 10^5^ cells/well. Once the cells reached 70–80% confluence, it was exposed to 10–50 *μ*M concentrations of LA or OxLA with or without pretreatment to PPAR antagonists, MK886 (10 *μ*M) or GW9662 (1 *μ*M). At the end of 4 or 12 hours of lipid treatment, RASMCs were rinsed with Hanks balanced salt solution (HBSS) followed by the addition of 5 × 10^4^ cells/cm^2^ THP1 (human monocytes) to each well. After 24 hrs coculture of RASMC and THP1, the plates were rinsed three times with PBS and adhered monocytes were counted in a 3 × 3 field under an inverted microscope (Leica-DMI4000B, Wetzlar, Germany) for each condition and duration of treatment. The average number of adhered monocytes for each treatment and time duration were counted by two independent investigators.

### 2.9. Statistical Analysis

The real-time PCR results were expressed as fold change ± Standard Error of ΔCt for each group compared to control using the Pfaffl method (2^−ΔΔCt^) [42]. All statistics were performed at the ΔCt stage in order to exclude potential bias due to averaging of data transformed through the equation 2^*∧*−(ΔΔCt)^[45]. One way ANOVA was used for the comparison between two treatments at each time point. Vehicle control of the baseline (no antagonist pretreatment) group was used as the control for all statistical comparisons. Differences due to treatments in the density of the protein bands after Western blotting and the number of attached THP1 monocytes were analyzed for significance by one-way ANOVA, compared to the untreated, vehicle control. The relative data was presented as mean ± Standard Error of mean. Significance was confirmed using post hoc analysis using Fisher's least significant difference (Fisher's LSD) test. A *P* < 0.05 was considered statistically significant. In the figures, significant differences between vehicle control and treated samples is indicated as an asterisk-*, whereas significant difference between the lowest concentration to higher concentrations of the lipid treatments is indicated as “^#^”.

## 3. Results

### 3.1. *ω*-6 Lipids Modulate PPAR Protein Levels and Transactivity

**Figure 1:**
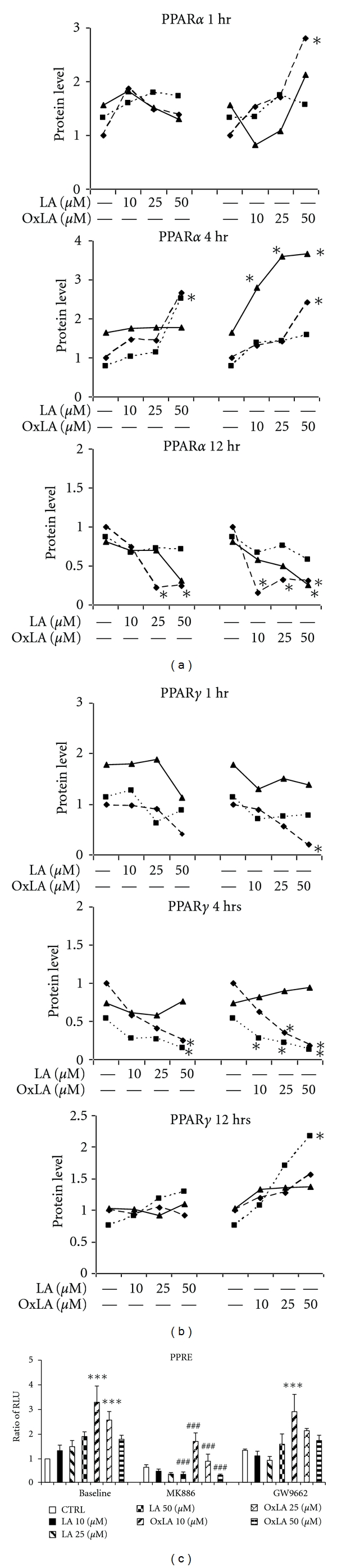
*ω*-6 lipids modulate PPAR protein levels and PPRE transactivity in a time-dependent manner. Western blotting of RASMC lysates treated with 10–50 *μ*M of LA and OxLA for 0–12 hrs using PPAR*α* and PPAR*γ* antibody shows induction of PPAR*α* at acute phase and PPAR*γ* at subacute phase. Control (CTRL) was defined as the cells treated with vehicle only. The results were expressed as mean ± SEM (Standard Error of Mean) defined by the ratio of protein levels in treated samples compared to CTRL. All data were normalized to *β*-actin (house-keeping protein) (a) PPAR*α* protein levels after 1 hr, 4 hr, 12 hrs treatment. (b) PPAR*γ* protein levels after 1 hr, 4 hr, 12 hrs treatment. The figure is a representation of three independent blots. One way ANOVA was used for the comparison between two treatments. Significance was confirmed using post hoc analysis using Fisher LSD test. *P < 0.05. (c) PPAR transactivity was measured in PPRE-luciferase transfected RASMCs which were pretreated with 10 *μ*M MK886 (PPAR*α* antagonist) or 1 *μ*M GW9662 (PPAR*γ* antagonist) followed by exposure to 10–50 *μ*M LA or OxLA for 4 hrs. The assay were run in duplicates and repeated three independent times. The results were expressed as mean relative luciferase activity ± SEM (Standard Error of Mean). One way ANOVA was used for the comparison between two treatments. Significance was confirmed using post hoc analysis using Fisher LSD test. *compared to CTRL, *P* < 0.05; ^#^compared to 10 *μ*M concentration, *P* < 0.05.

PPARs are transcription factors, which upon ligand activation, promote regulation of genes that exhibit PPAR response elements [46, 47]. The PPAR ligands regulate these transcription factors at the protein level. In RASMCs, LA and OxLA had a differential induction of PPAR subtypes, with an induction of PPAR*α* protein in the acute phase (1–4 hrs) (Figure 1(a)) and induction of PPAR*γ* at the subacute phase (12 hrs) (Figure 1(b)) compared to vehicle CTRL. The OxLA at increasing concentrations had 2–4-fold higher induction of PPAR*α* protein at 4 hrs but less than baseline levels at 12 hrs. In contrast, OxLA was less effective on PPAR*γ* protein, with an induction of only about 2-fold at 12 hrs. 

PPAR transactivity studies using RASMCs transfected with PPRE-luciferase constructs showed that compared to vehicle CTRL, both LA and OxLA induced PPRE transactivity in a concentration-dependent manner, (Figure 1(c)). Pretreatment of the cells with a PPAR*α* antagonist MK886, exhibited a significant attenuation of the PPRE activity that was induced at all concentrations of LA and OxLA (−81% for LA and −50–80% for OxLA) (*P* < 0.005) after 4 hrs treatment. On the contrary, pretreatment with PPAR*γ* antagonist, GW9662 only partially inhibited the PPRE transactivity induced by LA and OxLA (−17% for LA and −3–17% for OxLA), (Figure 1(c)). These results suggest a time-dependent modulation of PPAR subtypes by *ω*-6 lipids.

### 3.2. Ligand-Mediated Regulation of PPAR Transactivity Alters Egr-1/MCP-1 mRNA Levels

**Figure 2:**
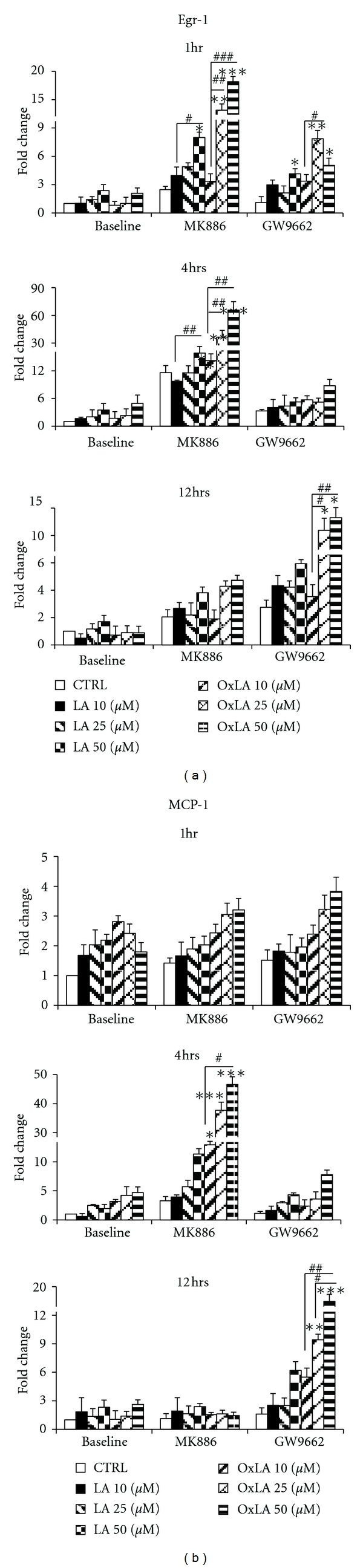
Ligand-mediated regulation of PPAR transactivity altered Egr-1/MCP-1 mRNA levels. Downregulation of PPAR*α* or *γ* by pretreatment of RASMCs with 10 *μ*M MK886 or 1 *μ*M GW9662 followed by exposure to 10–50 *μ*M LA or OxLA for 1–12 hrs resulted in time-dependent induction of Egr-1 and MCP-1 mRNA levels as analyzed using qRT-PCR. Control-(CTRL-) vehicle only. mRNA levels were expressed as fold change ± SEM (Standard error of ΔCT mean). (a) Egr-1 mRNA levels at 1, 4 and 12 hrs; (b) MCP-1 mRNA levels at 1 hr, 4 and 12 hrs. One way ANOVA was used for comparison between two treatments. Significance was confirmed using Fisher LSD test. *compared to CTRL, ^#^compared to 10 *μ*M concentration.

*ω*-6 lipids and its oxidized forms have dual effects on vascular cells. Since these lipids target both PPAR and Egr-1, we speculated that in the absence of PPARs (either by antagonizing the receptor using chemical antagonists or by siRNA approach), the *ω*-6 lipids will be able to activate Egr-1 and proinflammatory effects. The results shown in Figure 2 indicate that in the presence of a PPAR*α* antagonist MK886, the *ω*-6 lipids, LA, and OxLA had an immediate effect (acute phase) on Egr-1 and its downstream target MCP-1 mRNA levels. At 1 hour, both lipids significantly induced Egr-1 mRNA levels (3–20 fold) (Figures 2(a)-2(b)) but only had minimal effect on MCP-1 (1-2-fold), at all concentrations (10–50 *μ*M LA and OxLA) tested, compared to vehicle CTRL. This induction was further increased around 4 hrs, when levels of Egr-1 (10–80-fold) and MCP-1 (5–45-fold) by LA and Ox-LA was induced by 3–5 times higher than after 1 hr treatment and compared to vehicle CTRL. But around 12 hours, the Egr-1/MCP-1 levels returned to near baseline levels. Minimal effects on Egr-1/MCP-1 were observed by the antagonists themselves.

In contrast, in the presence of PPAR*γ* antagonist GW9662, the *ω*-6 lipids had a higher induction of Egr-1 and MCP-1 at a later time point, that is, 12 hours (subacute phase) (Figures 2(a)-2(b)). There was a minimal or no apparent induction of Egr-1 or MCP-1 at 1–4 hrs at all concentrations of LA and OxLA tested compared to vehicle CTRL. However, after 12 hours, cells exposed to GW9662 exhibited enhanced induction of Egr-1 and MCP-1 (5–10-fold) mRNA levels especially at higher concentrations of OxLA [18 fold, 50 *μ*M] compared to vehicle CTRL.

### 3.3. PPAR-Mediated Alteration in Egr-1/MCP-1 Protein Levels by *ω*-6 Lipids

**Figure 3:**
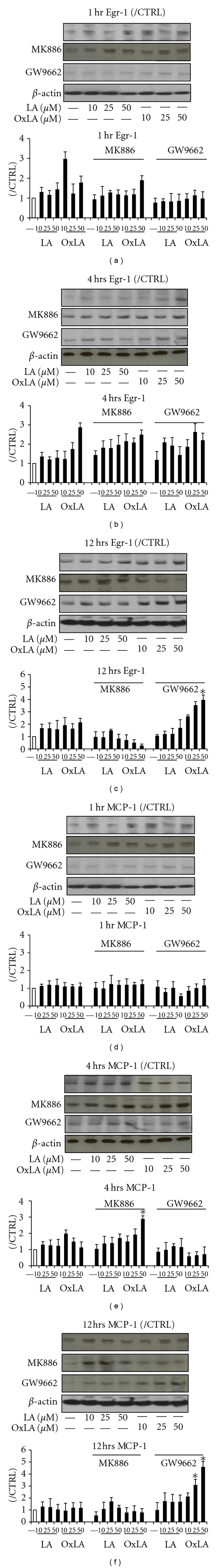
PPAR mediated alteration in Egr-1/MCP-1 protein levels by *ω*-6 lipids. Egr-1 and MCP-1 protein levels were determined using Western blot in cells exposed to *ω*-6 lipids after pretreatment with PPAR*α* or *γ* antagonists, MK886 or GW9662. Control-(CTRL) vehicle only. The results were expressed as mean ± SEM (Standard Error of Mean). (a–c) Egr-1 protein levels after 1, 4, and 12 hrs; (d–f) MCP-1 protein levels after 1, 4, and 12 hrs. One way ANOVA was used for the comparison between two treatments. Significance was confirmed using post hoc Fisher LSD test. *P < 0.05.

Western blotting of the cell lysates obtained from the above treated cells indicated that both LA and OxLA had similar trends on Egr-1 or MCP-1 protein levels as seen with the mRNA levels. As shown in Figures 3(a)-3(b), at 1 hr and 4 hrs (acute phase), LA and OxLA had minimal induction of Egr-1 protein. However, pretreatment with MK886 (PPAR*α* antagonist) exhibited a slightly larger induction of Egr-1 (2-fold). At 12 hrs the baseline Egr-1 protein levels were higher than that seen in the acute phase, however, when cells were pretreated with MK886 followed by exposure to *ω*-6 lipids there was a downregulation of Egr-1 protein levels, whereas pretreatment with GW9662, exhibited higher levels of Egr-1 protein, in a concentration-dependent manner (Figure 3(c)) compared to vehicle CTRL. This induction reached significance at 50 *μ*M OxLA (*P* < 0.05).

Similar trends were observed with the Egr-1 downstream target, MCP-1 protein levels in the presence of *ω*-6 lipids (Figures 3(d)–3(f)). MK886 pretreatment had an initial increase in MCP-1 levels at 4 hrs followed by a return to baseline levels at 12 hrs, upon exposure to *ω*-6 lipids. On the other hand, these lipids had minimal effects on MCP-1 protein levels when PPAR*γ* was inhibited (GW9662 pretreated cells) at all time points tested except for the higher doses of the OxLA at 12 hrs.

### 3.4. siRNA-Mediated Downregulation of PPARs Modulated Egr-1 Levels

**Figure 4:**
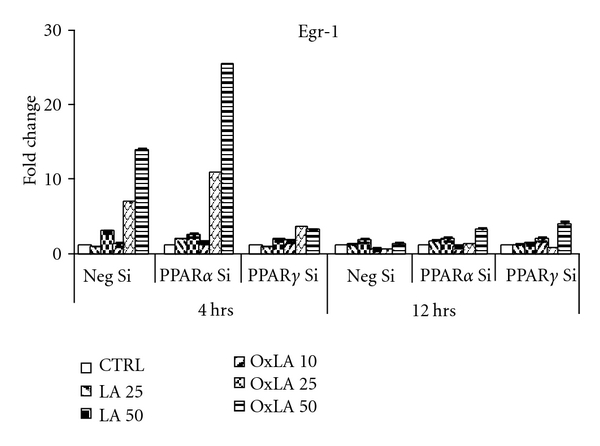
Downregulation of PPARs by siRNA alters *ω*-6 lipid-mediated induction of Egr-1 mRNA: RASMCs were transfected with SMART-pool siRNA duplexes of PPAR*α*, PPAR*γ* or nontargeting control siRNA, followed by treatment with *ω*-6 lipids for 4 and 12 hrs. Preliminary findings indicated Egr-1 mRNA levels were upregulated when PPAR*α* was knocked down compared to PPAR*γ* knockdown as determined by qRT-PCR. The fold change was calculated by comparing lipid treatments with vehicle controls in each siRNA group. All values were normalized to *β*-actin (house-keeping gene). mRNA levels were expressed as fold change ± SEM (Standard error of ΔCT mean).

The results above indicated that inhibition of PPARs by antagonists modulated Egr-1 levels by *ω*-6 lipids. In order to validate the above findings where PPARs were downregulated by the use of antagonists and establish the relationship between PPAR levels and Egr-1 modulation by *ω*-6 lipids, in this experiment we used siRNA approach to downregulate either PPAR*α* or PPAR*γ* in RASMCs followed by treatment with different concentrations of *ω*-6 lipids for 4 or 12 hrs. As shown in Figure 4, our preliminary findings indicated that compared to the concentration-dependent Egr-1 induction by *ω*-6 lipids in the nontargeting siRNA group, the Egr-1 levels were upregulated by over 20–25-fold when PPAR*α* was downregulated by siRNA approach at 4 hrs compared to PPAR*γ* downregulation. The effects of *ω*-6 lipids were less apparent at the subacute phase. These initial findings have similar trends in Egr-1 levels as observed in the presence of PPAR antagonists.

### 3.5. Alterations in Egr-1/MCP-1 Levels by *ω*-6 Lipids Modulates Monocyte Adhesion to RASMCs

**Figure 5:**
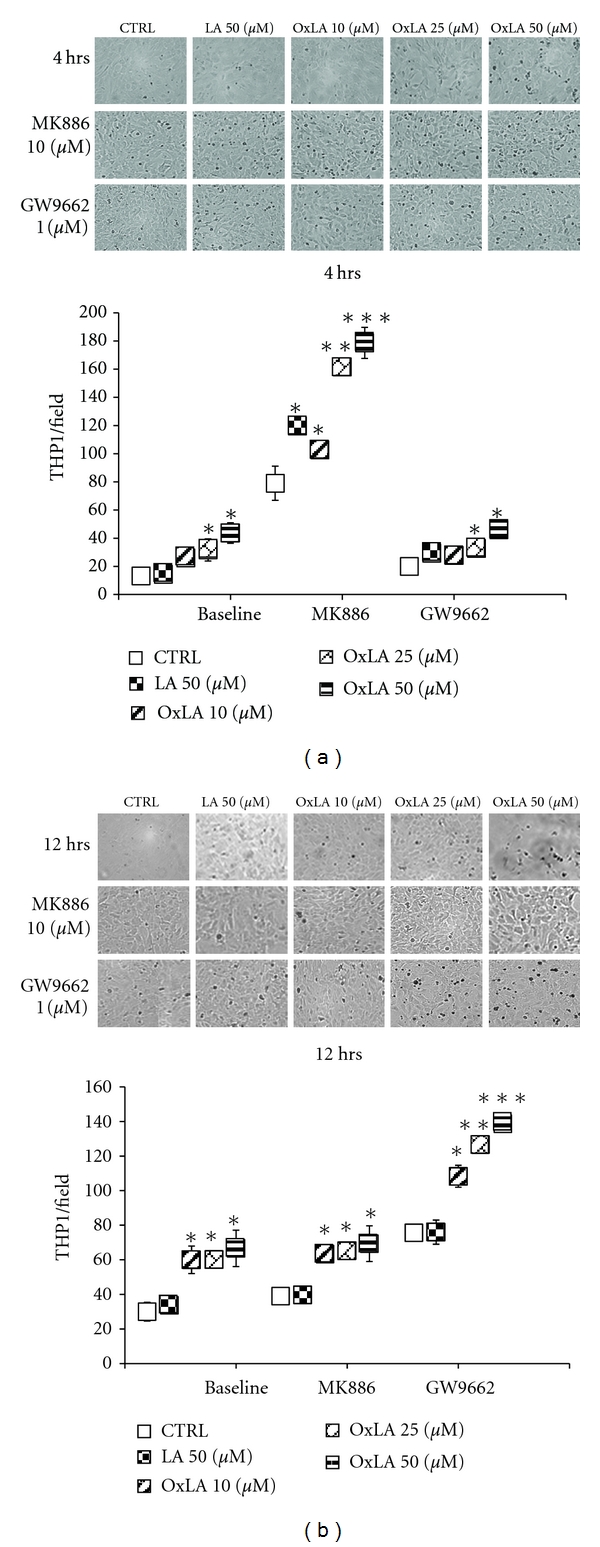
Alterations in Egr-1/MCP-1 by *ω*-6 lipids resulted in altered monocyte (THP1) adhesion to RASMCs THP1 (human monocyte) cells adhesion to RASMCs exposed to LA and OxLA (with or without pretreatment to MK886 and GW9662) was determined by counting the number of cells in a 3 × 3 field on an inverted fluorescent microscope. Results showed that monocyte adhesion reflected the alterations in Egr-1 and MCP-1 levels by *ω*-6 lipids by an increase in adherent monocyte cell number at 4 hrs when PPAR*α* was downregulated and increase in adherent cell number at 12 hrs when PPAR*γ* was downregulated. The data presented represents mean numbers of THP1 cells adhered to RASMCs in each field ± SEM (Standard Error of Mean). (a) monocyte adhesion at 4 hrs; (b) monocyte adhesion at 12 hrs. *P < 0.05, **P < 0.01, ***P < 0.005.

Monocyte/macrophage infiltration into the subendothelial space of arteries is an important step in the atherogenic process [48]. Egr-1/MCP-1 interplay plays an important role in promoting atherogenic lipids (OxLDLs) initiating monocyte infiltration and adhesion during atherosclerosis [49–51]. Since, our results thus far indicated that *ω*-6 lipids by regulating PPAR transactivity were able to modulate Egr-1/MCP-1 mRNA and protein levels, we speculated that this will alter monocyte adhesion to vascular cells in a time-dependent fashion. Figures 5(a)-5(b) demonstrate that in the absence of PPAR antagonists, *ω*-6 lipids had a concentration-dependent increase in the number of monocytes adhered to RASMCs (*P* < 0.05) at both 4 and 12 hrs treatments. However, in the presence of PPAR antagonists, these lipid ligands had a PPAR-subtype-dependent modulation of monocyte adhesion to RASMCs. Pretreatment with MK886 (PPAR*α* antagonist) significantly increased monocyte adhesion by PPAR lipid ligands at 4 hours, whereas PPAR*γ* inhibition (GW9662 treated) increased adhesion at a later time point (12 hours) reflecting the increase in Egr-1/MCP-1 mRNA and protein levels in the presence of these antagonists at these respective time points. PPAR antagonists by themselves did seem to have an effect on the monocyte adhesion to RASMCs, but the lipid treatments were in addition to what was observed at baseline.

## 4. Discussion

Our study for the first time demonstrates that atherogenic *ω*-6 lipids, such as linoleic acid and its oxidized forms (13-HPODE/13HODE), (abundant in diet and associated with OxLDL) modulate PPAR/Egr-1 crosstalk, resulting in altered vSMC function. In the presence of PPAR antagonists, *ω*-6 lipids altered Egr-1-mediated responses through its divergent effect on PPAR subtypes in a time-dependent manner. Though our studies used these lipids in the free form, in physiology these fatty acids are either part of membrane lipids or major components of lipoproteins. We predict that oxidation of most of the dietary lipids both in the free form or in an esterified form will have similar effects. Depending on its dose and time of exposure, *ω*-6 lipids have biphasic effects on vascular inflammation [51–53]. vSMCs that make up the intimal and medial layer of the vessel wall play an important role in the initiation and early progression of atherosclerosis [54]. *ω*-6 lipids mediate smooth muscle migration, proliferation, and apoptosis during the atherosclerotic process [55]. These lipids have multiple proinflammatory effects on the vasculature which include, activation of adhesion molecules, chemoattractants, NF*κ*B pathway, and activation of scavenger receptors leading to foam cell formation [50, 56, 57]. On the other hand, we and others have also shown anti-inflammatory effects of *ω*-6 lipids, such as their ability to inhibit tumor necrosis alpha (TNF*α*) production, inhibition of nitric oxide production, and activation of antioxidant enzymes [53, 58–60]. 

The zinc finger transcription factor, Egr-1, is expressed in all vascular cells including endothelial cells, smooth muscle cells, and monocyte/macrophages [61]. Egr-1 is also upregulated in the atherosclerotic fibrous cap [62]. Oxidative stress, an important player in atherosclerosis can induce Egr-1 [63, 64] and conversely, deletion of Egr-1 showed a protective effect in the Apo E^−/−^ atherosclerosis mouse model [65, 66]. Several factors including platelet-derived growth factor [67, 68], fibroblast growth factor [69], angiotensin-II [70], and oxidative stress [71] activates Egr-1 in vascular tissues including vascular smooth muscle cells [70]. A recent study demonstrated that oxidants such as H_2_O_2_, activated Egr-1 in vascular smooth muscle cells in both a time- and dose-dependent manner [64]. In the present study, in the presence of PPAR antagonists, the *ω*-6 lipids had a biphasic effect on PPAR subtypes, an activation of PPAR*α* in the acute phase (1–4 hours), and PPAR*γ* in the subacute phase (12 hours). The mechanism behind this biphasic effect can only presently be speculated to be a probable regulation of PPAR turnover by these lipids. *ω*-6 lipids are physiological ligands of PPARs and biophysical studies confirmed a covalent interaction between these lipids with PPARs [29, 30]. These interactions were different from that seen with known PPAR synthetic ligands that is, rosiglitazone or fibrates. In the present study, both from the preliminary PPAR siRNA findings and the PPRE-luciferase transactivity studies in RASMCs, in the presence of PPAR antagonists indicated that *ω*-6 lipids had a higher induction of PPAR*α* promoter compared to PPAR*γ*, however, we still observed that blocking PPAR*γ* did enhance Egr-1/MCP-1 in the subacute phase. This can probably be attributed to increased generation of intracellular oxidative stress including H_2_O_2_ [58, 72] at the subacute phase or through the inhibition of Egr-1 by PPAR*γ* [20, 23].

In both atherosclerotic and ischemic models, PPAR ligands are known to inhibit multiple proinflammatory genes by inhibiting Egr-1 [20, 21, 73]. Our findings further showed that the time-dependent effect of OxLA on PPARs/Egr-1 crosstalk and the resultant alterations in Egr-1/MCP-1 levels also resulted in altered smooth muscle cell chemoattraction to monocytes. Selective blocking of PPAR*α* (MK886) enhanced monocyte adhesion at 4 hrs, whereas blocking of PPAR*γ* (GW9662) enhanced monocyte adhesion at 12 hrs. This data further supports the time-dependent paradoxical effects of OxLA during the atherogenesis process [50, 53, 59]. Since, pretreatment with MK886 exhibited a higher inhibition of OxLA-induced PPRE transactivity but a higher induction of Egr-1/MCP-1 and monocyte adhesion than GW9662, it can be speculated that the *ω*-6 lipids seems to have a predominant influence on PPAR*α* compared to PPAR*γ* in vSMCs.

In the present studies, though both unoxidized and oxidized linoleic acid exhibited similar regulatory effects on Egr-1/MCP-1 and PPAR levels, at similar concentrations, OxLA had a more dramatic effect than LA. Both unoxidized and oxidized forms are ligands of PPARs and have been shown to have similar vascular effects. This may be explained by (i) the ability of LA similar to OxLA to generate reactive oxygen species (ROS), though at lower levels, by mitochondrial oxidation [74] and fatty acid peroxisomal degradation [75]. ROS generated through these pathways induces transcription factors including NF*κ*B and Egr-1 [71, 76, 77]. (ii) Secondly, other than dietary and other extracellular sources, the hydrolysis of esterified lipids by intracellular lipoxygenase and cycloxygenase pathway can also generate oxidized lipids [78, 79] from LA.

Our results, to our knowledge, for the first time demonstrate that *ω*-6 lipids depending on the dose and time of exposure on vascular cells have a preferential activation of specific PPAR subtypes. Whether this preferential activation of PPAR subtypes is reflective of the ability of these lipids to modulate PPAR turnover is currently being investigated. The interactions of these lipid ligands on both Egr-1 and PPAR subtypes results in an altered crosstalk between Egr-1 and PPARs which ultimately reflected in altered atherogenic response by the vascular cells (Scheme 1). Our results provide novel insights into the regulatory role of dietary *ω*-6 lipids on two of the major transcription factors that are relevant to atherosclerosis, PPAR, and Egr-1 with differing vascular effects.
